# GIWAXS experimental methods at the NFPS-BL17B beamline at Shanghai Synchrotron Radiation Facility

**DOI:** 10.1107/S1600577524004764

**Published:** 2024-06-25

**Authors:** Zhongjie Zhu, Lanlu Lu, Chunyu Li, Qingjie Xiao, Tingting Wu, Jianchao Tang, Yijun Gu, Kangwen Bao, Yupu Zhang, Luozhen Jiang, Yang Liu, Weizhe Zhang, Shuyu Zhou, Wenming Qin

**Affiliations:** a National Facility for Protein Science in Shanghai Shanghai Advanced Research Institute, Chinese Academy of Sciences Pudong District People’s Republic of China; University of Essex, United Kingdom

**Keywords:** synchrotron radiation, X-ray, GIWAXS, perovskite cells

## Abstract

The BL17B beamline at the Shanghai Synchrotron Radiation Facility was first designed as a versatile high-throughput protein crystallography beamline and one of five beamlines affiliated to the National Facility for Protein Science in Shanghai. It was officially opened to users in July 2015. As a bending magnet beamline, BL17B has the advantages of high photon flux, brightness, energy resolution and continuous adjustable energy between 5 and 23 keV. The experimental station excels in crystal screening and structure determination, providing cost-effective routine experimental services to numerous users. Given the interdisciplinary and green energy research demands, BL17B beamline has undergone optimization, expanded its range of experimental methods and enhanced sample environments for a more user-friendly testing mode. These methods include single-crystal X-ray diffraction, powder crystal X-ray diffraction, wide-angle X-ray scattering, grazing-incidence wide-angle X-ray scattering (GIWAXS), and fully scattered atom pair distribution function analysis, covering structure detection from crystalline to amorphous states. This paper primarily presents the performance of the BL17B beamline and the application of the GIWAXS methodology at the beamline in the field of perovskite materials.

## Abstract 2

The development of a versatile *in situ* grazing-incidence wide-angle X-ray scattering (GIWAXS) testing device at the BL17B beamline station at the Shanghai Synchrotron Radiation Facility in China is described.

## 1. Introduction

**Figure 1:**
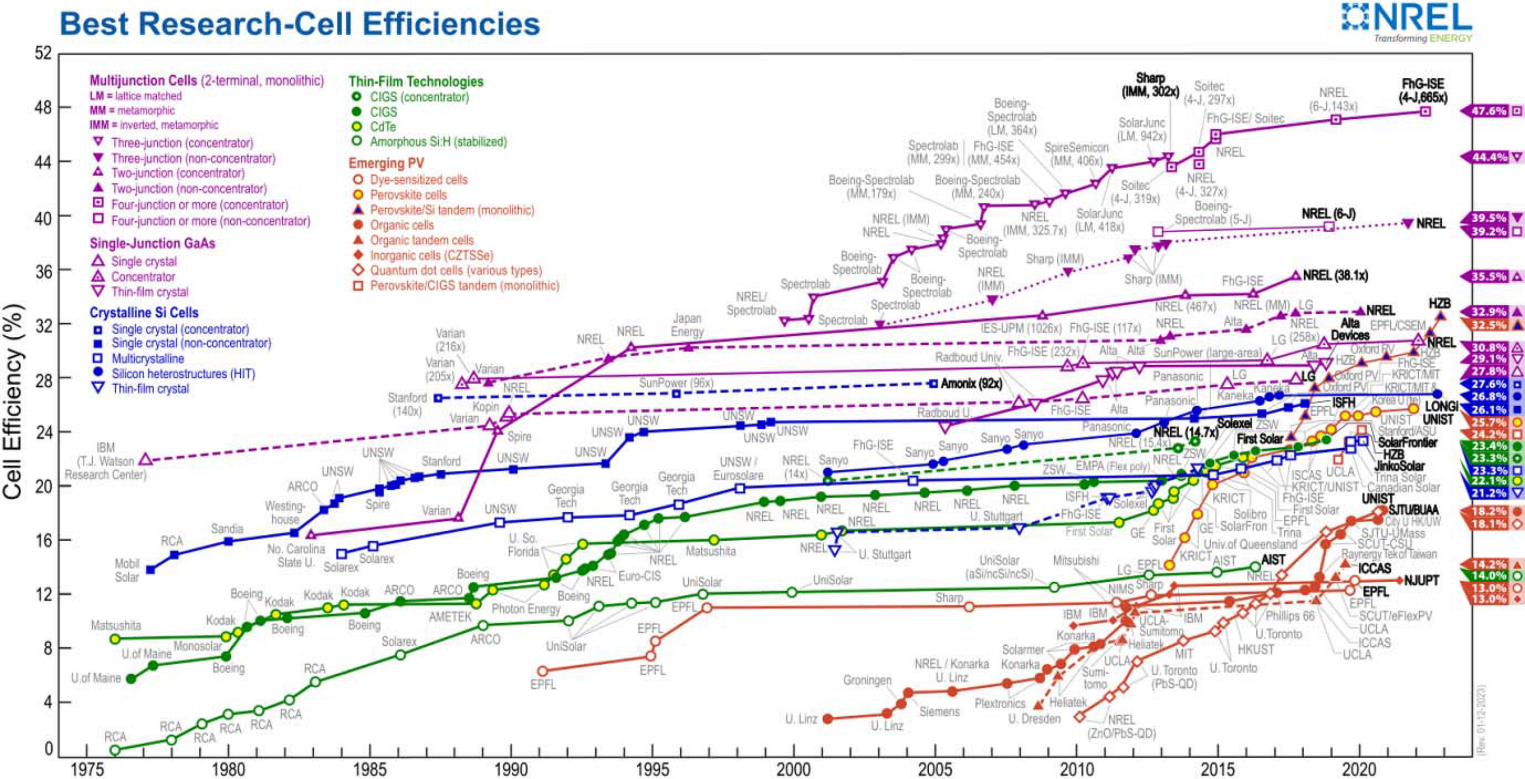
NREL shows the highest confirmed conversion efficiency for research cells used in a range of photovoltaic technologies from 1976 to the present.

Proteins play a crucial role in human life, composing cells and governing various aspects of life, impacting our health, development and survival. The ongoing quest by scientists and the medical community to comprehend the profound impact of proteins on human life is essential for advancing wellbeing and health. Concurrently, the field of materials science has made substantial contributions, particularly in energy materials development. According to the growing demand for efficient energy solutions, perovskite has emerged as a key material in battery and photocatalysis applications due to its exceptional electrical and light absorption properties. This has positioned perovskite as a pivotal player in advancing renewable energy technologies. The America’s National Renewable Energy Laboratory (NREL, https://www.nrel.gov/pv/cell-efficiency.html, see Fig. 1 ▸) has compiled certified efficiency data of various solar energy technologies, revealing remarkable progress of perovskite solar cells, with an efficiency of 26% (Zhao, Ma *et al.*, 2022 ▸).

In recent decades, perovskite cells have surpassed dye-sensitized solar cells and CdTe-based solar cells in production cost while maintaining high efficiency, gaining prominence in the solar cell materials field. In 2009, Kojima *et al.* (2009 ▸) used organic–inorganic hybrid perovskite materials for the first time to prepare perovskite dye-sensitized solar cells, achieving an efficiency of 3.8%. Since then, organic–inorganic hybrid perovskite solar cells have received much attention in the field of optoelectronics due to their excellent optoelectronic properties such as superior long carrier diffusion length and high optical absorption coefficient, and have broad prospects in optoelectronic applications (Ma *et al.*, 2022 ▸; Im *et al.*, 2011 ▸; Kim *et al.*, 2012 ▸; Lee *et al.*, 2012 ▸). In recent years, with the improvement of perovskite film material preparation methods and sample conditions, the photoelectric conversion efficiency of this type of material has been increasing year by year. (Burschka *et al.*, 2013 ▸; Jeon *et al.*, 2014 ▸; Zhou *et al.*, 2014 ▸; Yang *et al.*, 2015 ▸; Bi, Tress *et al.*, 2016 ▸; Bi, Yi *et al.*, 2016 ▸; Saliba *et al.*, 2016 ▸; Yang *et al.*, 2017 ▸; Jung *et al.*, 2019 ▸; Jiang *et al.*, 2019 ▸; Min *et al.*, 2019 ▸; Kim *et al.*, 2019 ▸; Yoo *et al.*, 2021 ▸). Until 2022, You and co-workers (Zhao, Ma *et al.*, 2022 ▸) converted PbI_2_ into an inactive (PbI_2_)_2_RbCl compound by RbCl doping, which effectively stabilizes the perovskite phase. They obtained a certified power conversion efficiency of 25.6% for FAPbI_3_ (where FA is formamidinium) perovskite solar cells on the basis of this strategy. As a new photoelectric material with high efficiency, low cost and convenient preparation, the performance of perovskite battery material is closely related to its crystal structure and morphology. However, perovskite materials have complex compositions and variable phases, and more advanced characterization techniques are urgently needed to study their internal structural changes and microscopic mechanisms. Characterizing material structure is crucial for a comprehensive understanding of material properties and performance. Synchrotron X-ray diffraction (XRD) technology, with advantages such as high intensity and high resolution, allows for in-depth exploration of the crystal structure and crystallographic properties of crystalline materials. This advanced technique provides a key means to fully comprehend material structure characteristics. Particularly for perovskite thin film materials, grazing-incidence wide-angle X-ray scattering (GIWAXS) technology is indispensable.

**Figure 2:**
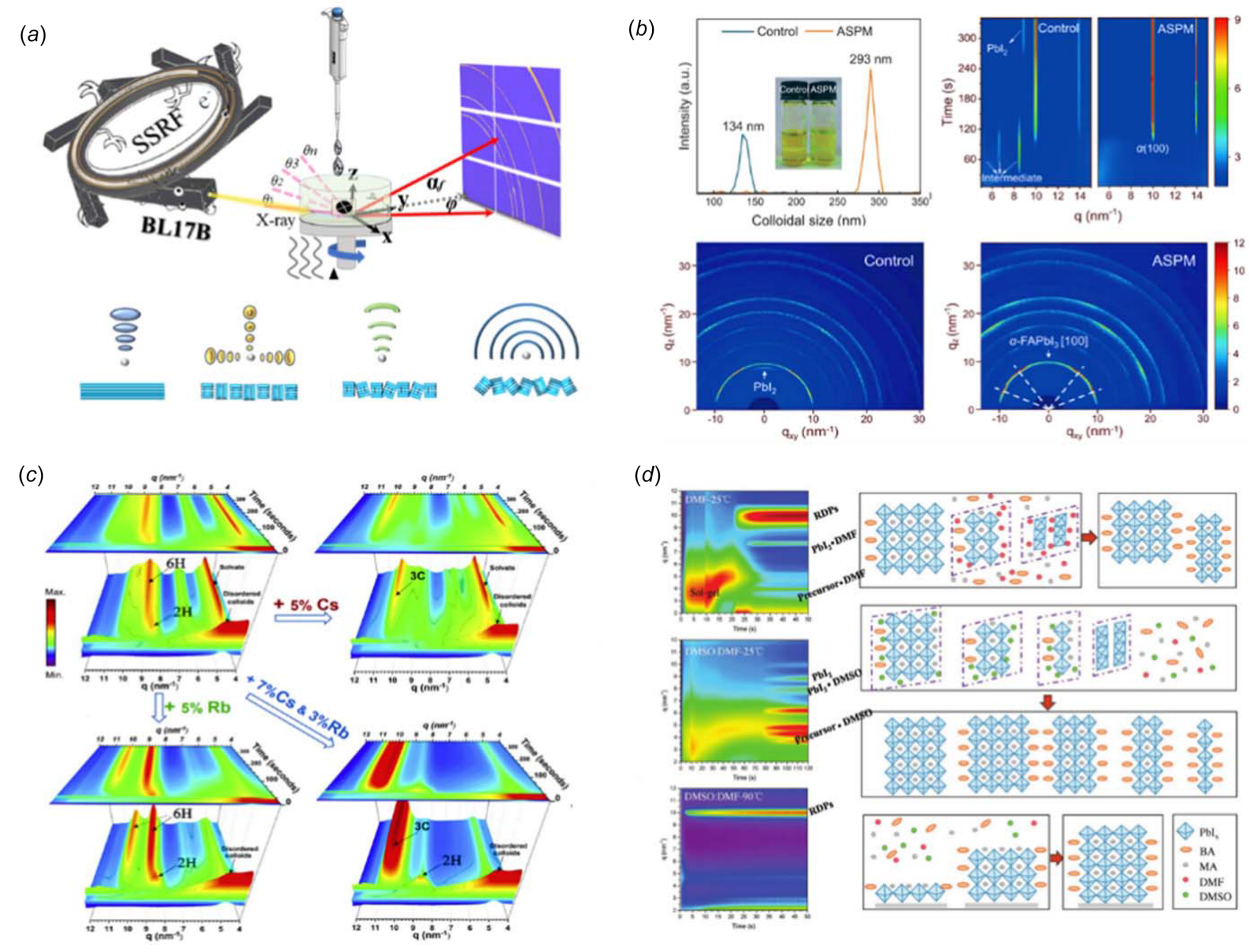
(*a*) Schematic diagram of GIWAXS on the BL17B beamline at the SSRF. (*b*) The nucleation and crystallization process of perovskite films (Zhu *et al.*, 2024 ▸). (*c*) Cs^+^ and Rb^+^ addition dictate the growth of perovskite films (Dang *et al.*, 2019 ▸). (*d*) *In situ* GIWAXS measurements and schematic models showing the reduced-dimensional hybrid perovskite (RDP) formation for the three distinct fabrication conditions (Zhang *et al.*, 2018 ▸).

GIWAXS based on synchrotron X-rays is a powerful analytical tool for the study of perovskite solar cells. GIWAXS data typically consists of two-dimensional diffraction patterns of a diffraction ring with different crystal planes (Wang *et al.*, 2021 ▸), which is widely used in the field of perovskite research because of its advantages such as high signal-to-noise ratio, high structural resolution, non-contact, non-destructive detection, rich structural information, depth resolution and *in situ* observation. It provides information about the crystal structure, crystallinity, grain size and orientation of the perovskite material in thin films, which is crucial for understanding the material’s performance and stability. It can be used to study the formation and evolution of perovskite grains during the fabrication process, as well as the effect of processing conditions on grain size, orientation and crystallinity. This information can help optimize the fabrication process to produce high-quality perovskite thin films with improved performance and stability. This method can also be used to study the interface between the perovskite material and other layers in the solar cell, such as the electron transport layer and the hole transport layer. Understanding the interface structure and composition can provide insight into the charge transfer and transport processes in the solar cell, and help identify ways to improve their efficiency. In summary, GIWAXS technology is an important tool for the study of perovskite solar cells and provides valuable information for understanding the material properties and optimizing their performance. Above all, *in situ* GIWAXS measurements are highly attractive in perovskite research as they offer a fast way to track the microstructural changes of perovskite materials, including crystallization and aging processes. This is crucial for revealing the dynamic processes of perovskite materials. The film formation process involves a liquid film gelation stage and a crystallization stage. Many studies have shown that the orientation and phase structure of perovskite are established in the gelation stage. The *in situ* GIWAXS technique for real-time monitoring of the perovskite crystallization process can be traced back to as early as 2015, when Miyadera *et al.* (2015 ▸) analyzed *in situ* the crystallization kinetics of CH_3_NH_3_PbI_3_ perovskite at SPring-8, Japan, by X-ray diffraction immediately after combining PbI_2_ thin film with CH_3_NH_3_I solution. In 2018, Zhang *et al.* (2018 ▸) investigated the film formation mechanism and its dependence on choice of solvent and substrate temperature by performing *in situ* time-resolved GIWAXS at the Cornell High Energy Synchrotron Source (CHESS) during spin coating. In 2020, Hoffman *et al.* (2020 ▸) used *in situ* GIWAXS to monitor film formation during spin-coating, elucidating the general film formation mechanism of 2D halide perovskites during one-step spin-coating at the Advanced Photon Source in Argonne National Laboratory, USA. In the same year, Song *et al.* (2020 ▸) measured the crystal structure and orientation of perovskite films prepared with and without KI and DMSO additives based on the GIWAXS technique at the Advanced Light Source (ALS), Lawrence Berkeley National Laboratory, USA. In the past three years in particular, a number of research groups have explored the structural mechanism of perovskite solar thin film cells based on the Shanghai synchrotron radiation light source, which has promoted the improvement of the conversion efficiency of multiple types of cells (Jiang, Sun *et al.*, 2022 ▸; Zhao, Ma *et al.*, 2022 ▸; Yang *et al.*, 2023 ▸; Liang *et al.*, 2023 ▸). The *in situ* methodology and some *in situ* experimental results are shown in Fig. 2 ▸.

**Figure 3:**
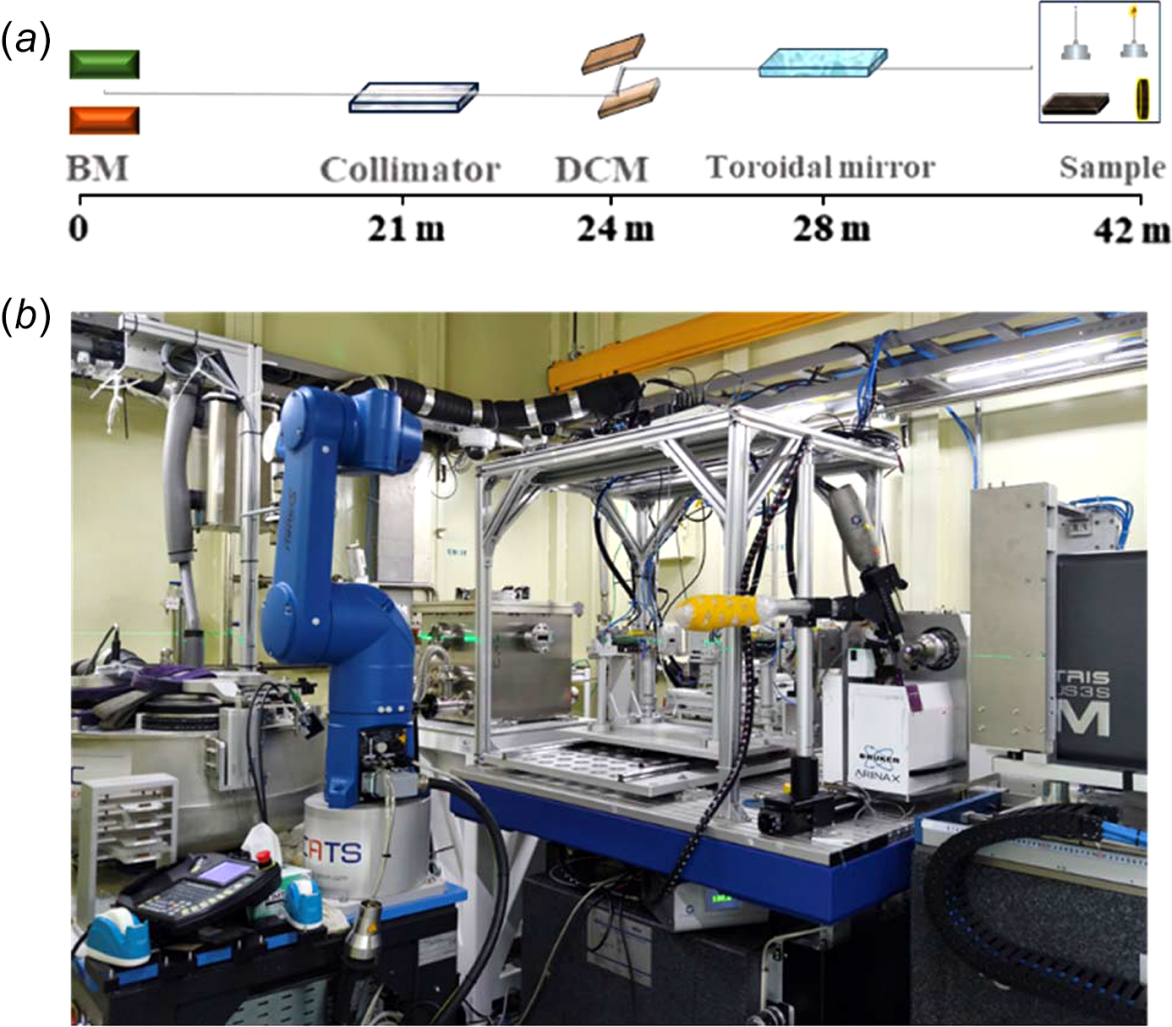
The standard bending magnet front-end layout of SSRF.

Currently, synchrotron beamlines both domestically and internationally, such as SPring-8 BL46XU, ALS 7.3.3, Shanghai Synchrotron Radiation Facility (SSRF) BL14B and BL17B, and CHESS D-line, are capable of conducting GIWAXS methodological studies. In comparison with others, SSRF-BL17B not only possesses the capability for batch testing but also integrates various *in situ* experimental conditions significantly, including heating, spin-coating, different atmospheric environments, and dropwise solvent addition, among others. The BL17B high-throughput protein crystallography beamline station consists of a bending magnet source, front-end, optical elements, experimental station and sample predation area. The photon source of the beam is a bending magnet light source, which is based at the SSRF. The electron energy of the storage ring is 3.5 GeV, and the natural divergence is 3.9 nm rad. The magnetic field intensity is 1.2726 T and the bending radius is 9.1673 m. The station uses a standard SSRF bending magnet front-end to achieve high-throughput beamlines. The beamline is 42 m long from the source to the sample position [see Fig. 3 ▸(*a*)]. A photograph of the experiment station for XRD is shown in Fig. 3 ▸(*b*).

The beam design uses the pre-collimator as the first optical element. The collimating mirror is a bent mirror for vertical focusing to enhance the energy resolution. Also, it is used to cut off the high-order harmonics. To eliminate the high-order harmonics, the surface of the mirror will be divided into two tracks parallel to the X-ray direction. One of the tracks has no coating and the other track is coated with rhodium. At a setting of 2.8 mrad, the uncoated track is used for photon energies below 8 keV and the Rh-coated track for energies in the range 8–23 keV. These settings will reject the higher-order harmonics. The high-order harmonics (mainly third harmonics) over base harmonics will be below 10^−3^ when we use a Si surface within the energy range between 5 and 8 keV and use a Rh surface within the energy range between 8 and 23 keV. For reference, the characteristics of this bending magnet source and relevant parameters are detailed in Table 1 ▸. Properties of the beamline BL17B are given in Table 2 ▸.

The BL17B beamline station utilizes synchrotron radiation as the X-ray source, offering advantages such as high flux, high collimation and tunable wavelength. Equipped with a two-dimensional area Pilatus detector, the experimental station not only rapidly acquires crystallographic information but also detects diffraction signals both in-plane and out-of-plane, providing detailed orientation distribution information for the same crystal plane. This capability facilitates time-resolved *in situ* experiments. By altering the incident angle, scattering signals from the surface to the bulk can be obtained. Therefore, experiments employing the synchrotron-radiation-based GIWAXS methodology have significantly contributed to the advancement of energy storage materials.

### 1.1. Experimental station

**Figure 4:**
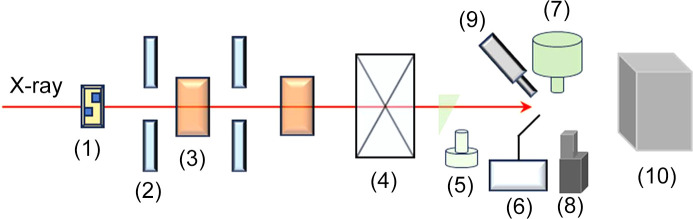
Layout of the experimental station: (1) attenuator, (2) slit, (3) ion chamber, (4) fast shutter, (5) co-axis microscope, (6) robot, (7) goniometer, (8) fluorescence detector, (9) cryocooler, (10) Pilatus detector.

The experimental hutch offers room to house all the required equipment, *e.g.* beryllium window, quadrant beam-position monitor, attenuator, slits, ion chamber, fast shutter, micro-diffractometer, Pilatus detector (3S 2M). Additionally, supplementary components such as sample alignment microscopes, sample cryocoolers, fluorescence detectors and more are available. The layout of the experimental station is shown in Fig. 4 ▸.

The MD2 diffractometer system boasts a high-precision air-bearing rotation axis with a programmable controller, enabling the issuance of shutter commands based on the rotation axes’ speed and position. The comprehensive beamline control system integrates slits, light intensity detectors and scatter blockers, among other components. External attachments to the diffractometer include a liquid nitro­gen cooling apparatus and an adjustable bracket for fluorescence detection. The low-temperature cooling equipment for the sample consists of a cold head, a liquid nitro­gen dewar, a pump, a cold head controller, a liquid nitro­gen level controller and a manual controller. The cold head’s adjustable temperature range spans from 80 to 500 K (Oxford Cryostream 800 series), facilitating *in situ* studies of temperature effects on the sample. A user-friendly goniometer head allows for easy manual installation of a standard test sample, enhancing operational efficiency.

The Pilatus 3S 2M detector is classified as a medium-sized area detector, and employs water cooling to maintain low temperatures (https://www.dectris.com/en/support/manuals-docs/pilatus3-x-for-synchrotron/pilatus3-x-2m/). This detector offers high frame rates for shorter data collection times, and increasing efficiency. Simultaneously, it possesses the advantage of minimal noise background, allowing it to record stronger diffraction signals and minimizing the risk of overexposure. Detailed specifications for this detector are presented in Table 3 ▸.

During the data collection process, the motion of all goniometer motors is controlled by the MD2 control software, which enables real-time monitoring of camera and sample center positions. Additionally, this software allows for the selection of beam apertures to modify the beam size and offers functions for controlling the shutter, sample loop automatic centering and beam alignment. The MD2 control software communicates with the motors through the PMAC control system. Data acquisition for the Pilatus 2M detector is managed by Camserver (Zhang *et al.*, 2019 ▸). All hardware control software is coordinated through the *Macromolecular Xtallography Customized Beamline Environment* (*MXCuBE*) system (Oscarsson *et al.*, 2019 ▸). The *Experimental Physics and Industrial Control System* (*EPICS*) is employed to govern the motion of all motors for optical and support systems. It is also used to retrieve signals from beamline devices following protocols specified by SSRF. The beamline station features a 20 TB capacity storage server for data storage. Due to storage limitations, diffraction images are currently retained on the server for one month. Furthermore, commonly used crystallography software includes *HKL3000*, *XDS*, *APEX4*, the *CCP4* suite, *PHENIX*, *SHELX*, *etc*. Commonly used powder, film and other test data processing software include *Fit2d* (Hammersley, 1997 ▸), *Dioptas* (Prescher & Prakapenka, 2015 ▸), *pyFAI* (Ashiotis *et al.*, 2015 ▸), *GIWAXS-tools* (Pan *et al.*, 2021 ▸) and so on.

**Figure 5:**
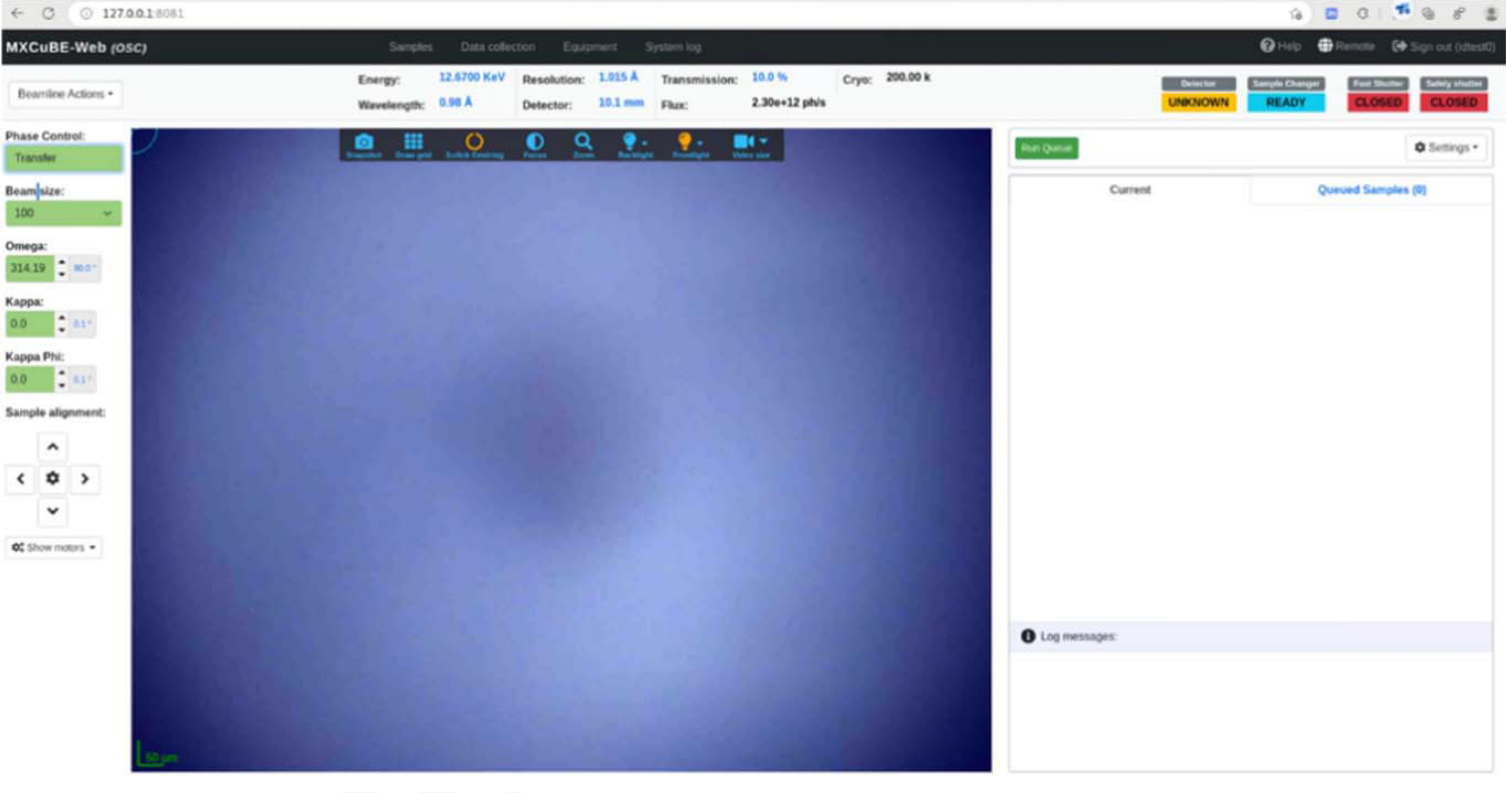
Schematic diagram of the *MXCuBE* software user interface.

In order to improve the performance of the beamline station and protect the equipment in the experimental hutch, an air conditioning system is equipped to ensure that the temperature in the experimental hutch is 25 ± 1°C. The humidity in the laboratory chamber is controlled by an industrial dehumidifier and the reading humidity control system has been upgraded. In 2022, the BL17B data acquisition system was replaced with the *MXCuBE* system. The development of this system can be traced back to 2004 when it was initially co-developed by the European Synchrotron Radiation Facility (ESRF) and other partners. Its primary aim was to provide a comprehensive tool for simplifying and automating the process of data collection in macromolecular crystallography. *MXCuBE* has been made available as an open-source platform, encouraging participation from the research community and customization as needed. Over time, *MXCuBE* has undergone multiple versions of improvements and updates, including added functionalities, enhanced user interfaces, expanded detector support and performance optimizations. This tool has found widespread use globally, serving not only at ESRF but also at various other international synchrotron facilities. It is worth noting that, due to the evolving nature of this field, *MXCuBE* may have undergone further developments and improvements. This system is a customizable experimental control system designed specifically for macromolecular crystallography data collection. It provides automated control, a user-friendly interface, real-time data analysis, data storage and management, as well as support for various X-ray detectors. *MXCuBE* accommodates various experimental plans, such as standard data collection, rapid acquisition and high-throughput strategies, with the aim of offering convenient tools for researchers in macromolecular structural biology, expediting the data collection and analysis process, thus advancing the study of molecular structures. The specific functionalities of *MXCuBE* may vary depending on the laboratory or beamline, and an example of its user interface is shown in Fig. 5 ▸.

## 2. GIWAXS methodology of BL17B

### 2.1. Sample preparation

**Figure 6:**
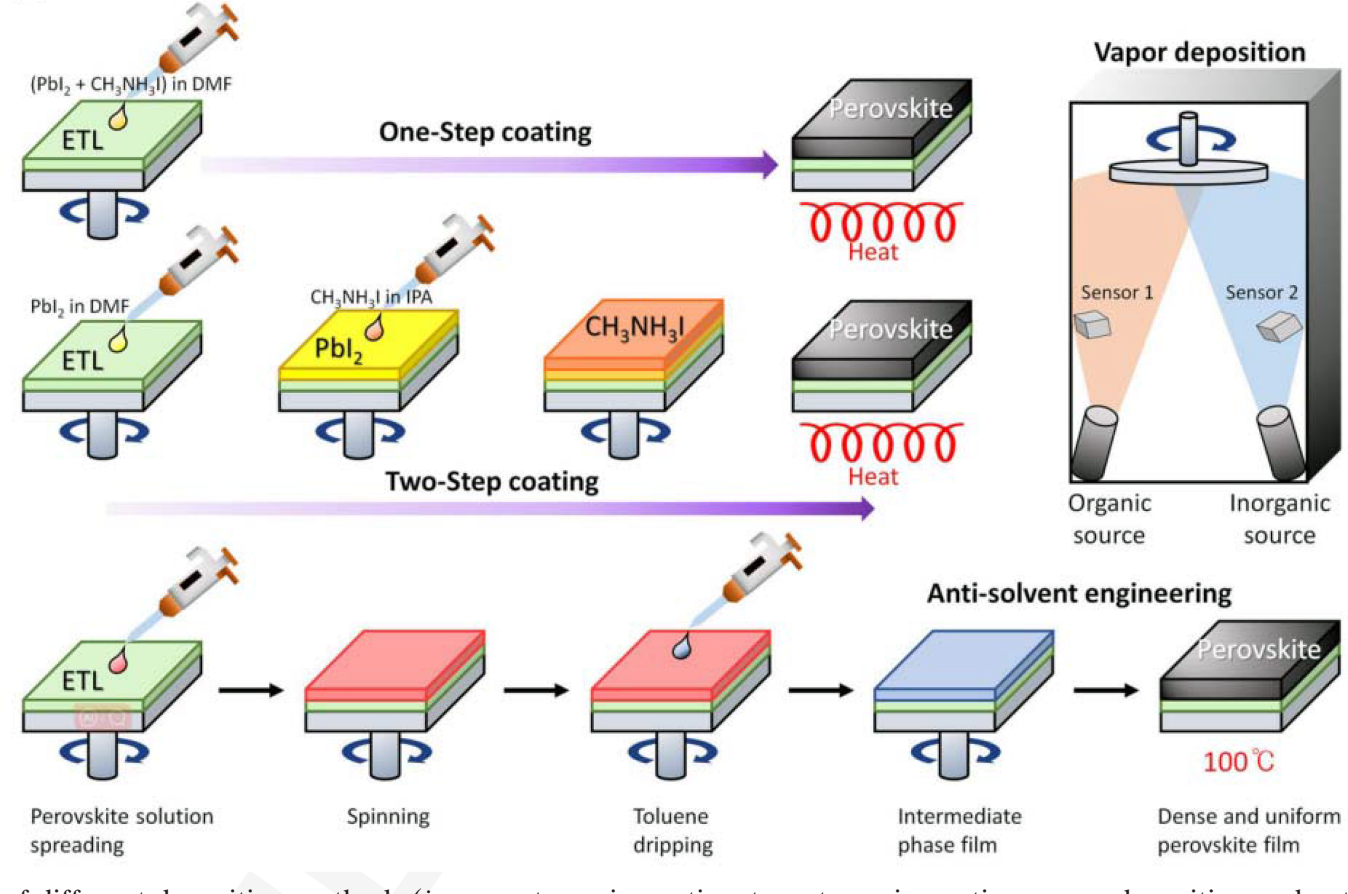
Schematic illustration of different deposition methods (*i.e.* one-step spin-coating, two-step spin-coating, vapor deposition and anti-solvent engineering) (Choi *et al.*, 2020 ▸).

Typically, samples for GIWAXS testing are thin films grown on substrates, and there are various methods for preparing thin film materials like perovskite solar cell films. The key to preparing this material lies in the perovskite layer. In addition to the intrinsic optoelectronic properties of the material itself, the microstructure of the perovskite thin film also significantly affects the optoelectronic performance of the solar cell. This impact includes factors like light absorption, charge diffusion distance and lifetime, carrier transport and recombination, all of which are influenced by the morphology of the active layer thin film. An ideal perovskite crystal thin film should be dense, free of pinholes and have large and uniformly distributed grains. Such a film is crucial for achieving high photovoltaic performance in solar devices. To obtain high-quality perovskite crystal thin films, various film fabrication methods have been attempted, with the most commonly used methods being one-step solution deposition, two-step solution deposition and vapor deposition (Choi *et al.*, 2020 ▸) (see Fig. 6 ▸). Furthermore, the exploration of fabrication processes for highly crystalline, uniform and dense perovskite thin films is a prerequisite for achieving high-efficiency photovoltaic devices, and it is considered a crucial step towards the large-scale commercial production of perovskite solar cells in the future. Apart from the three perovskite thin film preparation methods mentioned earlier, there are a multitude of other methods, especially for large-area films, such as blade coating techniques (Xiao *et al.*, 2021 ▸), spray coating (Heo *et al.*, 2016 ▸), magnetron sputtering (Gao *et al.*, 2021 ▸), hybrid chemical vapor deposition (Leyden *et al.*, 2014 ▸) and more.

BL17B experimental users can select the most appropriate preparation method based on the characteristics of their samples. For *in situ* sample testing, it is recommended that the sample substrate area does not exceed 2 cm × 2 cm, the substrate is typically a silicon wafer or a glass slide, and the sample on the substrate should be uniformly coated with a thickness on the micro-nanometre scale.

### 2.2. Experimental method

**Figure 7:**
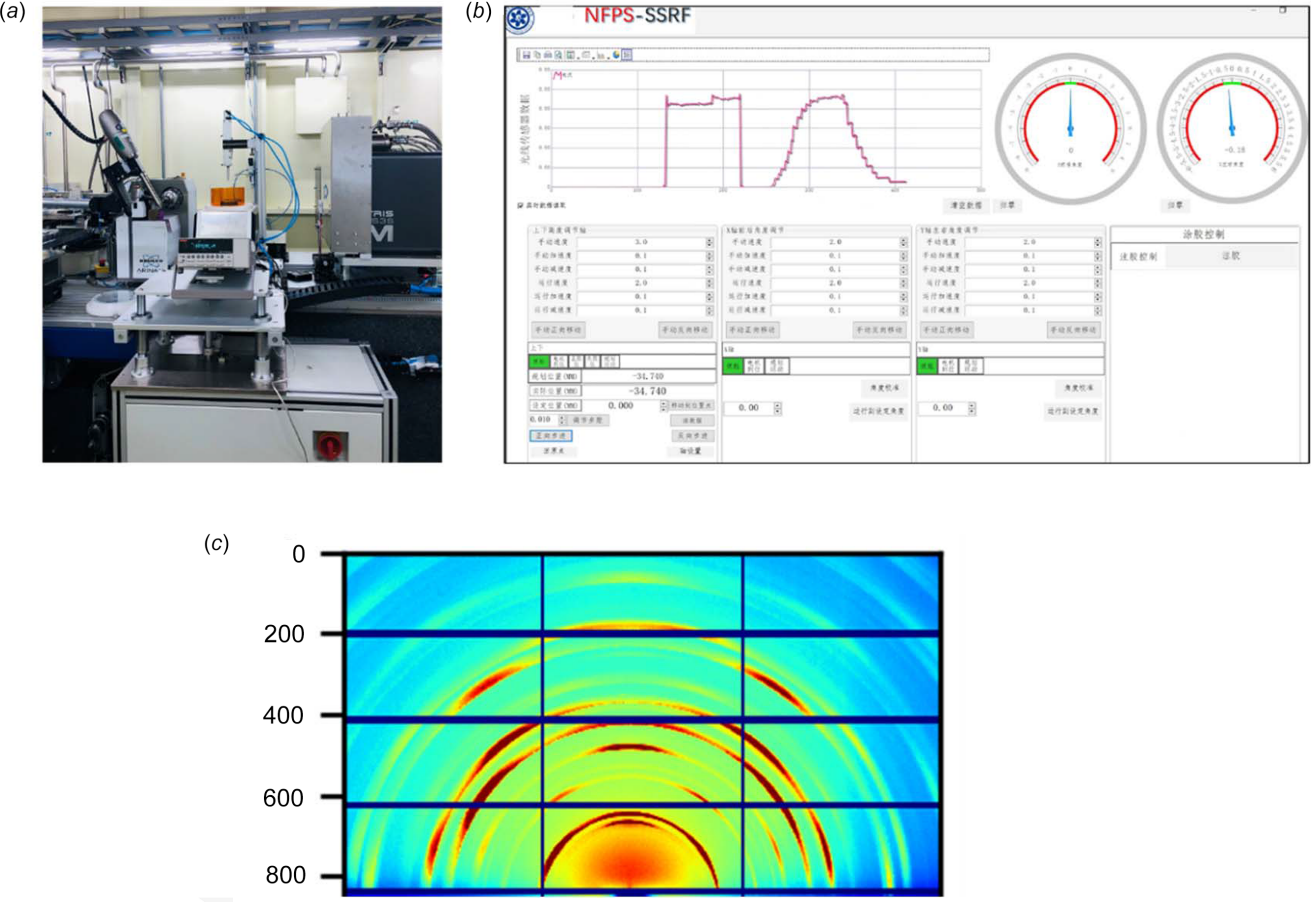
(*a*) Photograph of the portable control station and (*b*) diagram of the software user interface. (*c*) An example of a diffraction pattern recorded in the GIWAXS experiment.

The multifunctional GIWAXS testing apparatus at SSRF’s BL17B beamline, complete with hardware controllers and software, is depicted in Figs. 7 ▸(*a*) and 7(*b*), which also showcase a photograph and software interface. It comprises four main components: the support system, motion control system, sample multi-environmental variable system and signal reception system. The support and motion control systems offer multi-directional mobility to accurately position the device between the X-ray beam and the detector during experiments. Linear motors on the support system facilitate vertical movement of the sample on the support platform along the *Z*-axis (the *Z*-axis has a travel range of 290 mm, adjustable with a precision of 0.01 mm per step), ensuring precise X-ray exposure to the sample. The X-rays are monitored through readings from photodiodes and picoammeters in the signal reception system. Additionally, the motion control system can adjust the tilt angle of the electric turntable to precisely control the incident angle and penetration depth of the X-rays, meeting various sample requirements (the *Y*-axis has a range from 0 to 8°, with an adjustable increment of 0.1° per step). This system allows us to adapt flexibly to different experimental needs, enabling detailed research into sample crystalline behavior. The multi-environmental variable system for samples can be precisely configured according to user requirements, including, but not limited to, parameters such as temperature, spin-coating speed, atmospheric conditions, and more. This system’s flexibility and adjustability empower researchers to exercise comprehensive control and fine-tuning to match various experimental objectives and material characteristics, ensuring the optimal match of experimental conditions and obtaining accurate and reliable results. Finally, a user-friendly interface is designed to facilitate users in easily configuring experimental parameters and monitoring experiment progress. The remote-control system should possess the capability for remote operation, facilitating remote experimentation and data acquisition. Fig. 7 ▸(*c*) illustrates an example of data testing results during the calibration of the GIWAXS equipment.

Currently, the GIWAXS testing methods at the BL17B beamline are categorized into offline experiments and *in situ* experiments. Offline experiments refer to testing pre-prepared samples directly on the sample stage in the laboratory. Our designed sample stage can accommodate ten samples at once for automatic testing, significantly enhancing testing efficiency compared with the traditional method of testing individual samples sequentially. The method described in this paper greatly improves the testing throughput. In addition, *in situ* experiments encompass *in situ* spin-coating, heating and variations in environmental atmosphere. When *in situ* spin-coating and heating tests are required, the multifunctional control station is positioned between the X-ray beam and the detector, and the sample to be tested is securely placed and fixed on the sample stage. The motion control system and the spin-coating and heating device are used to accurately adjust the platform to achieve the desired X-ray exposure on the sample. The spin-coating and heating system is a fixture on the motion system, capable of heating the spin coater. The sample is placed on the spin head, either mechanically secured or attached with specific adhesives to ensure full contact with the spin head without obstructing incident and diffracted light.

**Figure 8:**
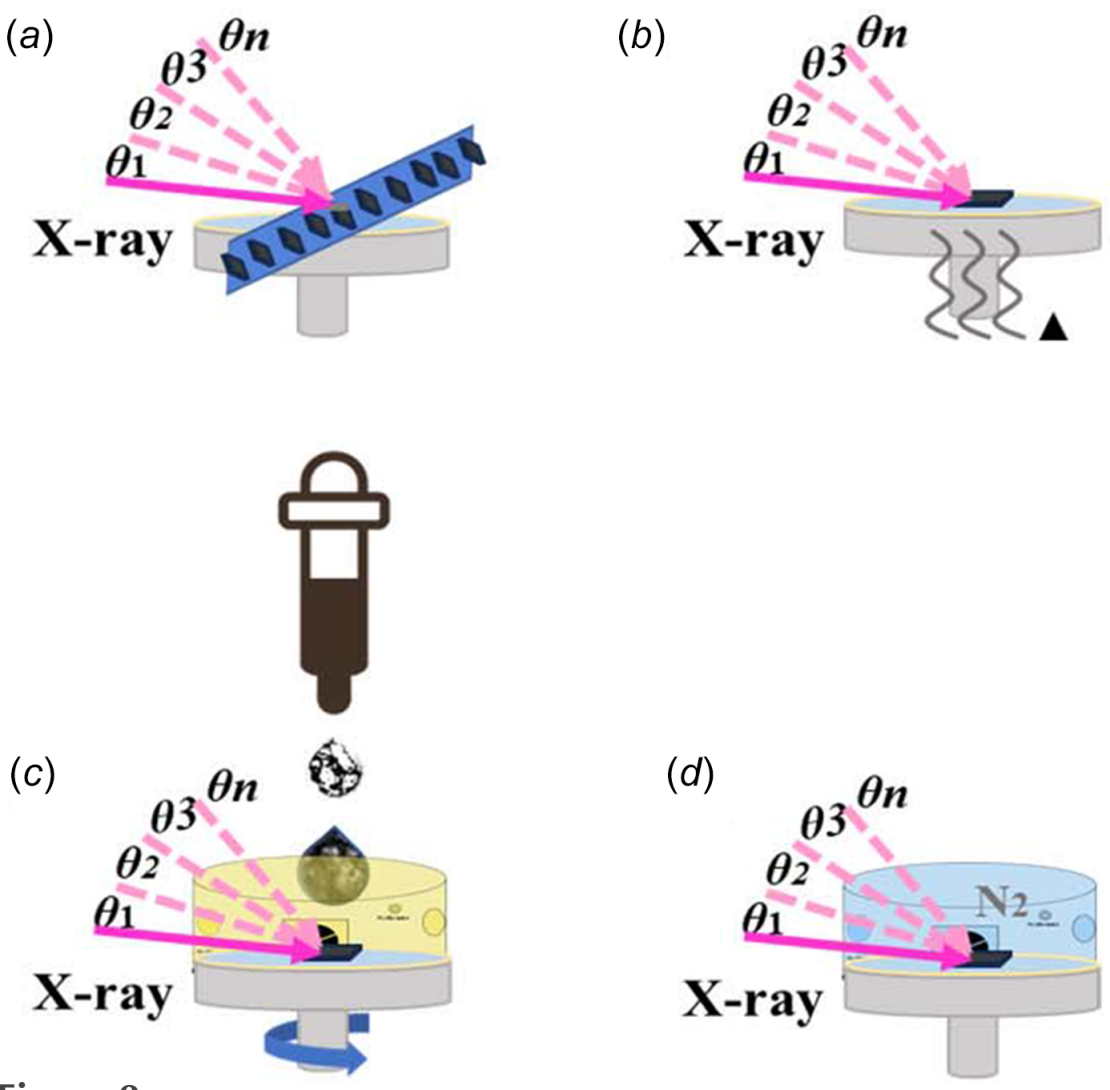
Schematic diagram of *off situ* and *in situ*. (*a*) *Off-situ* test, (*b*) *in situ* heating, (*c*) *in situ* spin coating and (*d*) *in situ* atmosphere environment.

The spin-coating heating device utilized at our beamline is a customized adaptation of commercial equipment, enabling remote operation. The maximum rotation speed of the spin-coating and heating device is 5000 r.p.m., with a rotation speed resolution of 1 r.p.m. The longest time for a single rotation is 50 min, and the rotation time resolution is 0.1 s. The maximum acceleration is 5000 r.p.m. s^−1^. The temperature can be elevated from room temperature to 423 K (heating time: 5 min), with a heating plate temperature uniformity of ≤±2%. Cooling can be assisted by the cold head. Additionally, another spin-coating apparatus is also remotely controllable, with a maximum rotation speed of 9999 r.p.m., maximum acceleration of 9999 r.p.m. s^−1^, rotation speed accuracy of ±1 r.p.m. and rotation speed resolution of 1 r.p.m. If additional liquids need to be added during the spin-coating and heating process, this can be done remotely through a pipetting gun. The pipetting gun is controlled by a pneumatic pump. If it is necessary to maintain a specific atmospheric environment during the spin-coating and heating process, an atmospheric hood can be placed on top of the spin-coating and heating device. This hood has two through holes in the direction of the light path. Since the incident X-ray beam is narrow while the diffracted X-ray beam is broader, the diffracted beam hole needs to be much larger than the incident beam hole. To ensure the atmosphere during spin-coating, the through holes and the sample outlet need to be sealed with Kapton film during usage. The atmospheric hood is equipped with intake and exhaust vents at both ends. During experiments, the intake vents should be connected to the required gas pipeline (such as nitro­gen), and the desired gas should be continuously supplied throughout the testing period. An illustrative diagram of the experimental method is provided in Fig. 8 ▸. The specific equipment parameters are presented in Table 4 ▸.

In GIWAXS testing, beam cutting is the first and most crucial step. Beam cutting refers to aligning the sample parallel to the X-ray beam and partially blocking the beam to ensure that the light can impinge on the sample surface in a grazing incidence manner during testing. Firstly, the sample is positioned lower than the beam so that the detector fully receives the beam, resulting in the highest detector count. In the second step, the sample position is raised along the *Z*-axis until the detector count decreases to half of the previous count, indicating that half of the beam is blocked. Finally, the sample is oscillated around the *Y*-axis within a small angular range. It can be observed that, when the sample is perfectly parallel to the beam, the beam is least obstructed, resulting in the maximum detector count. This angle is set as 0°, and afterward the incident angle can be controlled by tilting the sample stage. For testing perovskite thin film samples, an incident angle of 0.3° is typically chosen, as it achieves a good balance between diffraction signal and background signal. If it is necessary to limit the penetration depth, the incident angle can be adjusted accordingly. Additionally, the penetration ability of X-rays is closely related to their wavelength. Beamline BL17B usually employs X-rays with two wavelengths, 10 keV and 18 keV. The use of 18 keV X-rays provides a higher signal-to-noise ratio in the two-dimensional diffraction pattern, but it also has a higher penetration power and is less sensitive to changes in incident angle. Therefore, when performing angle-resolved tests, 10 keV X-rays are typically selected.

### 2.3. Scientific highlights of GIWAXS

**Figure 9:**
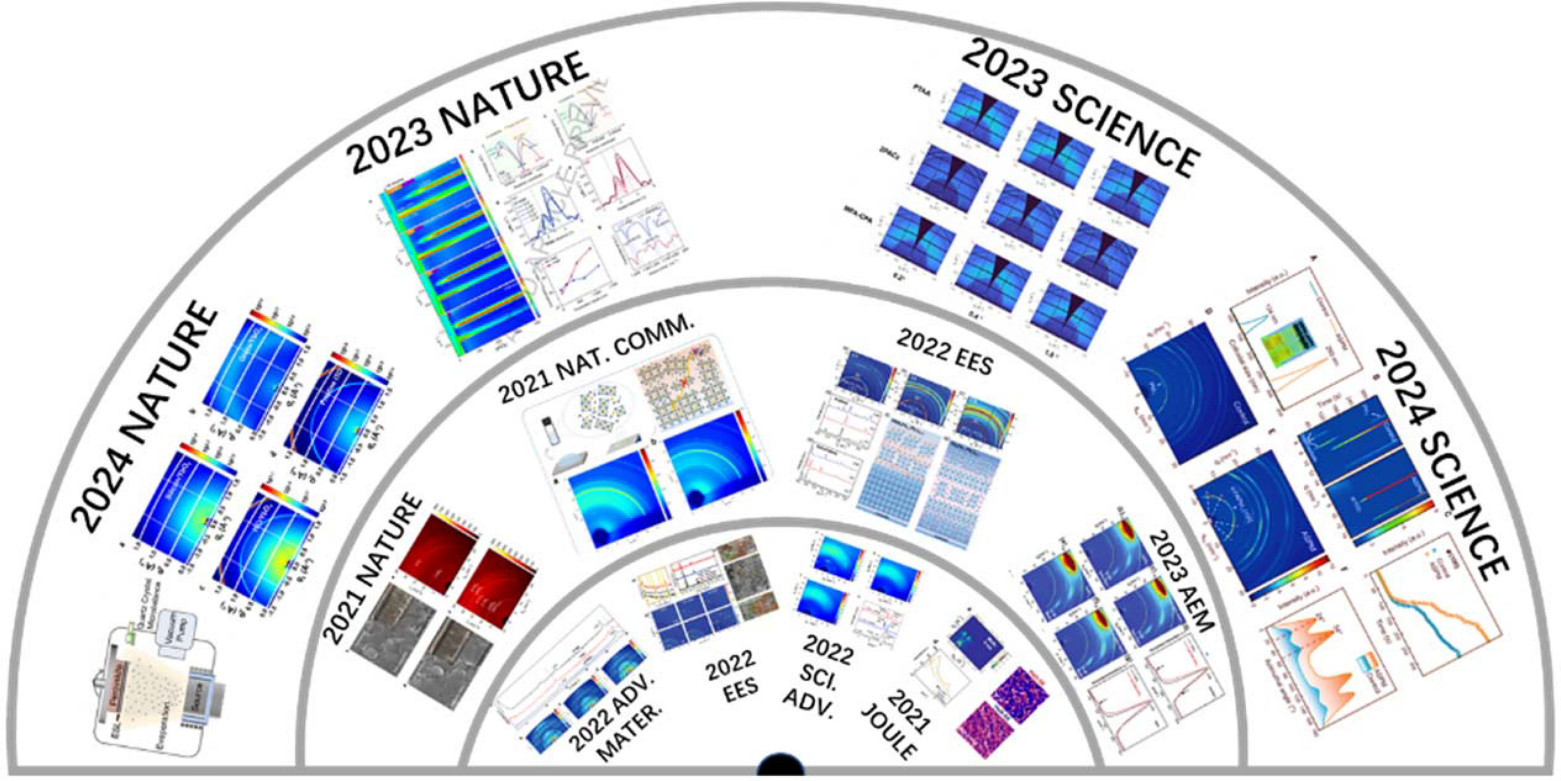
Typical achievements in GIWAXS research at the BL17B beamline.

Since 2015, beamline BL17B has served more than 400 research groups and 900 research topics, achieved fruitful results, and published a number of cutting-edge scientific research papers. These publications have a significant impact on perovskite research and serve as important references for potential users conducting GIWAXS studies on the BL17B beamline (Zhang *et al.*, 2021 ▸; Li *et al.*, 2020 ▸; Lu *et al.*, 2020 ▸; Wang *et al.*, 2020 ▸; Zong *et al.*, 2020 ▸; Jeong *et al.*, 2021 ▸; Jia *et al.*, 2021 ▸; Kong *et al.*, 2021 ▸; Li *et al.*, 2021 ▸; Ma *et al.*, 2021 ▸; Niu *et al.*, 2021 ▸; Xiang *et al.*, 2021 ▸ ;Yang *et al.*, 2021 ▸; Jiang, Chu *et al.*, 2022 ▸); Jiang, Sun *et al.*, 2022 ▸; Chen *et al.*, 2022 ▸; Lu *et al.*, 2022 ▸; Zhao, Ma *et al.*, 2022 ▸; Yu, Pavlica *et al.*, 2022 ▸; Yu, Meng *et al.*, 2022 ▸; Zhao, Zhang *et al.*, 2022 ▸; Yang *et al.*, 2023 ▸; Liang *et al.*, 2023 ▸; Chen *et al.*, 2024 ▸) (see Fig. 9 ▸)

In recent years, research scholars have conducted studies utilizing the GIWAXS testing apparatus at beamline BL17B, achieving significant breakthroughs and repeatedly setting world records in energy conversion efficiency. For instance, Pan research group (Liang *et al.*, 2023 ▸) devised a strategy utilizing 1-(phenyl­sulfonyl)­pyrrole to achieve a homogeneous distribution of cation composition within perovskite thin films. The resulting p-i-n device yielded a certified steady-state photon-to-electron conversion efficiency of 25.2% and enduring stability. This study was conducted at the *in situ* synchrotron beamline BL17B, utilizing *in situ* spin-coating and heating methods coupled with grazing-incidence wide-angle X-ray scattering (GIWAXS) to investigate two key kinetic processes – crystallization and phase transition – during the formation of perovskite materials. It reveals that the introduction of perovskite precursor solution accelerates both of these processes. Compared with other synchrotron beamlines, the GIWAXS testing at BL17B offers advantages in terms of rapid data collection speed as well as simple and portable remote operation. These studies have shown that excellent battery performance can be obtained by adjusting the uniformity of the out-of-plane distribution of perovskite components. Compared with other beamlines, BL17B boasts outstanding features as a bending magnet light source line. Its characteristics include high photon flux, brightness and energy resolution, with tunable energy ranging between 5 and 23 keV. Within the experimental hutch, there is ample space between the diffractometer and detector, facilitating the setup of GIWAXS workstations. The GIWAXS methodology developed at this beamline integrates offline and *in situ* experiments, addressing issues encountered at other beamlines such as narrow energy ranges or challenges with certain *in situ* experiments. The GIWAXS technology takes a crucial step forward in advancing the research and development of perovskite solar cells in academia and industry.

## 3. Conclusion

This article outlines the performance of the BL17B beamline station and the methodology of GIWAXS, including experimental testing, auxiliary facilities, data acquisition and user achievements. BL17B is a high-throughput structural analysis beamline with a high degree of automation, enabling rapid, scalable and efficient sample collection and structural determination. A portable/integrated/high-precision GIWAXS testing device developed based on this beamline allows for various *in situ* experiments, such as *in situ* coating, heating and atmospheric conditions. This device facilitates real-time monitoring of the crystallization and decomposition processes of perovskites, providing valuable support for the development of more stable and efficient perovskite solar cells.

## Figures and Tables

**Table 1:** Characteristics of the source and parameters

Energy (Gev)	Natural emittance (nm rad)	Magnetic field (T)	Bend radius (m)	Parameters of the souce			
				σ_*x*_ (µm)	σ_*x*_′ (µrad)	σ_*y*_ (µm)	σ_*y*_′ (µrad)
3.5	3.9	1.2726	9.1673	70	114	22	1.97

**Table 2:** Beamline details

Beamline	BL17B
Source type	Bend magnet (BM)
Collimating mirror	2.8 mrad Rh/Si Si/Si
Focusing mirror	2.8 mrad Rh/Si
Monochromator	Double-crystal monochromator (DCM), Si(111)
Energy range	5–23 keV, 2.5–0.6 Å
Energy resolution (Δ*E/E*)	≤3 × 10^−4^ (12.4 keV)
Focused beam size (FWHM)	≤120 µm × 180 µm (H × V, 12 keV)
Focused beam divergence	≤1.5 mrad × 0.2 mrad (H × V, 12 keV)
Flux	≥3 × 10^11^ photons s^−1^ (300 mA, 12 keV)
Goniometer	ARINAX MD2 Mini-Kappa MK3
Cryocapability	80–400 K, LN_2_ Oxford Cryosystem 800
Sample mounting robot	Irelec CATS
Detector	Pilatus3S 2M

**Table 3:** Pilatus S3 2M detector parameters

Name	PILATUS 2M
Type	3 × 8 CMOS
Area (mm)	254 mm × 289 mm
Pixel size	172 µm × 172 µm
Number of pixels	1475 × 1679
Dynamic range	1048576
Readout time	0.0023 s
Highest resolution (Å)	0.83 Å

**Table 4:** Relevant parameters of experimental devices Note: all of the equipment listed in the table can be operated by remote control. Power supply used: voltage AC220V (±10%), power 150 W.

Equipment	Parameter	
Spin coater (vac-sorb)	Maximum speed	9999 r.p.m.
	Precision index	±1 r.p.m.
	Single maximum time	50 min
Heating apparatus	Temperature control range	Room temperature ∼423 K
	Effective thermal area	π × 2.5^2^ cm^2^
Atmosphere hood	Shield	π × 6^2^ × 18 cm^3^
	Available gas	N_2_, CO, *etc.*
Pipette	Eppendorf	100 µL/200 µL/1000 µL
